# Surgical Simulation Course for Facial Fracture Education

**DOI:** 10.1097/GOX.0000000000003353

**Published:** 2021-01-25

**Authors:** Shannon S. Wu, Anooj Patel, Brendan Alleyne, Garyn Metoyer, Archana Chandrashekar, Bashar Hassan, Kshipra Hemal, Raffi Gurunian

**Affiliations:** From the * Cleveland Clinic Lerner College of Medicine, Cleveland, Ohio; † Case Western Reserve University School of Medicine, Cleveland, Ohio; ‡ Department of Plastic Surgery, Cleveland Clinic, Cleveland, Ohio; § Wright State University, Boonshoft School of Medicine, Dayton, Ohio; ¶ Midwestern University Arizona College of Osteopathic Medicine, Glendale, Ariz.; ∥ Wake Forest School of Medicine, Winston-Salem, N.C.

## Abstract

A hands-on facial fracture simulation course can be an important adjunct teaching modality in resident education and training, enhancing both resident confidence and competence in treatment of facial fractures. In this study, 11 plastic surgery residents participated in a surgical wet laboratory and lecture focusing on operative management of facial fractures. Pre- and post-course questionnaires were administered as clinical knowledge assessments. Pre-course, 40% of participating residents reported feeling comfortable with facial fracture management (>5 of 10) and 50% of residents achieved competence on clinical assessment (scoring >50%). Following the simulation course, these same assessments were re-administered. Post-course, comfortability with fracture management increased to 100% among participating residents, and 90% of residents scored >50%, demonstrating improvement in clinical competency.

## Abstract 2

Supplemental Digital Content is available in the text.

## INTRODUCTION

Residency training in non-tertiary centers with limited craniomaxillofacial trauma exposure requires supplemental education.^1^ Cadaveric simulation is the gold standard in surgical education, given its high fidelity to living tissue and anatomy. Yet, no cadaveric models for complex facial fractures are easily reproducible or widely accessible. A reproducible cadaveric model can train residents on operative facial fracture management, with deliberate creation of unique injuries, advanced decision-making in the presence of pedagogic faculty oversight, and enhanced exposure in non-trauma centers.

This study describes our experience with an innovative adaption of an age-old technique, by conjoining a cadaver-based laboratory with lecture to teach residents operative management of complex facial fractures. Intentional creation of precise and specific maxillofacial fractures using an osteotome in cadaveric specimen provides plastic surgery trainees representative clinical scenarios that would otherwise not be available. To test this model’s efficacy, we administered questionnaires to assess residents’ competence and confidence with facial fracture management before and after the course.

## METHODS

The course was developed by the senior author (RG, Director of Maxillofacial Trauma) using a grant from Stryker. The course objective was to improve residents’ assessment and operative management of midface and mandibular fractures, focusing on the principles of exposure, reduction, and fixation.

**Fig. 1.**
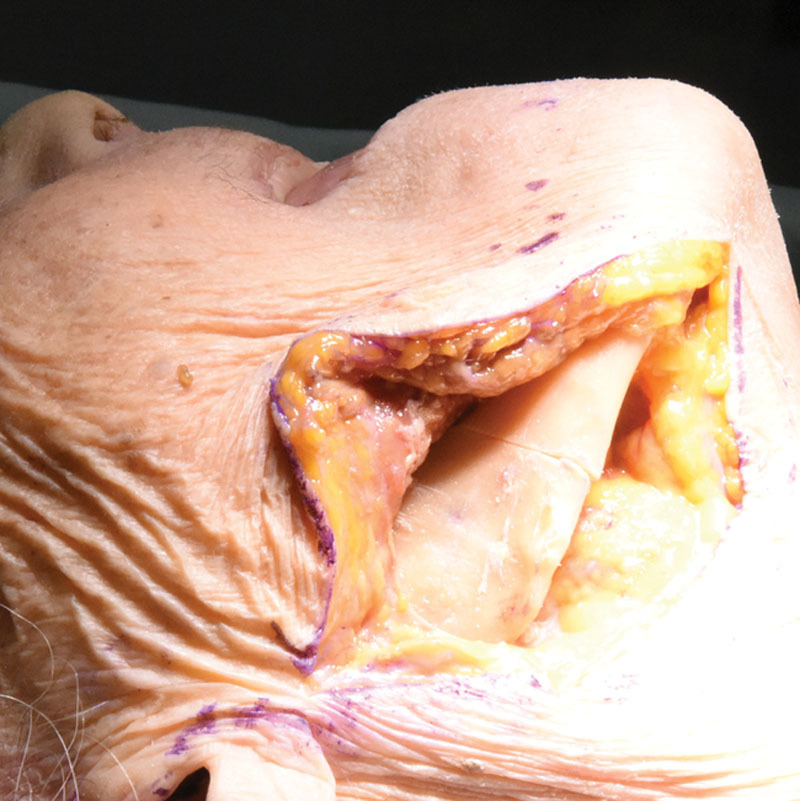
Submandibular approach and simulation of a body fracture using an osteotome.

**Fig. 2.**
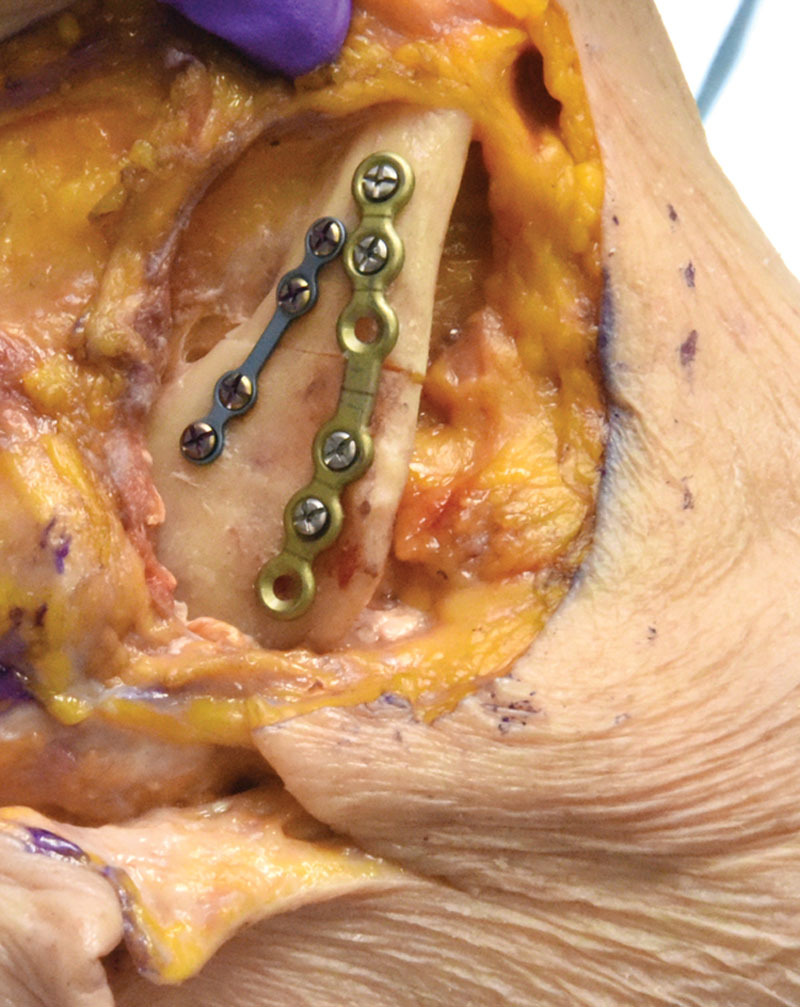
Fixation of the fracture using inferior plate and bicortical locking screws and superior plate (tension band) using monocortical locking screws.

Surveys were administered for course evaluation; thus, IRB approval was waived. In total, 11 plastic surgery residents from postgraduate year (PGY)-1 to PGY-6 participated. The course comprised a didactic lecture and hands-on cadaver simulation. An osteotome was employed to simulate comminuted and displaced craniofacial fractures in fresh and frozen cadaver heads (Fig. 1). Complex midface, zygoma, and frontal sinus fractures (including gingival sulcus, coronal, eyebrow, and lower eyelid) were designed. Compared with prior studies that used random blunt force to the entire head to result in the facial fracture,^2^ the Stryker module purposefully created fractures conceptualized ahead of time. Residents exercised operative approaches with oversight from attending plastic surgeons and technical assistance from Stryker representatives. Approaches to mandibular fractures included submandibular (Fig. 2), intraoral, retromandibular, and pre-auricular. Approaches to orbital fractures included subciliary, transconjunctival, and transcaruncular. All residents attempted and practiced these different approaches in the 8-hour session.

The same questionnaires were administered pre- and post-course. Residents were asked their postgraduate year level and confidence level in the individual steps of operative fracture management on a scale from 1 to 10, with 1 signifying “not at all comfortable” and 10 signifying “very comfortable” (**See survey, Supplemental Digital Content 1**, which displays the questionnaire administered to all participants before and after Facial Fracture Education Course. http://links.lww.com/PRSGO/B554). Comfort was defined as >5 on a 10-point scale. An in-service competence assessment was administered pre- and post-course. The effectiveness of the course was measured by mean change in correct answers on the competence assessment and self-reported comfortability, as measured by paired *t*-test.

## RESULTS

**Fig. 3.**
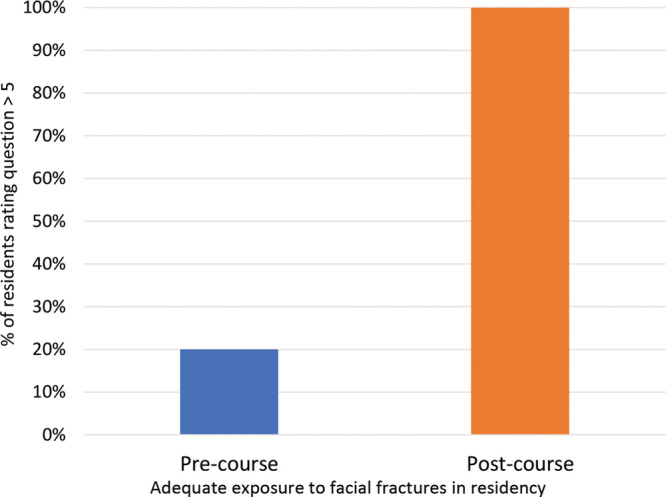
Resident response when asked about adequate exposure to facial fractures in residency before and after the Facial Fracture Education Course on a scale of 1–10, with 1 signifying “not at all comfortable” and 10 signifying “very comfortable” with adequate exposure to facial trauma in thus far in residency training.

Eleven residents completed the questionnaire and competence assessment before and after the course. Pre-course, 20% of residents were comfortable with their exposure to facial fractures, which increased to 100% after the course (Fig. 3).

**Fig. 4.**
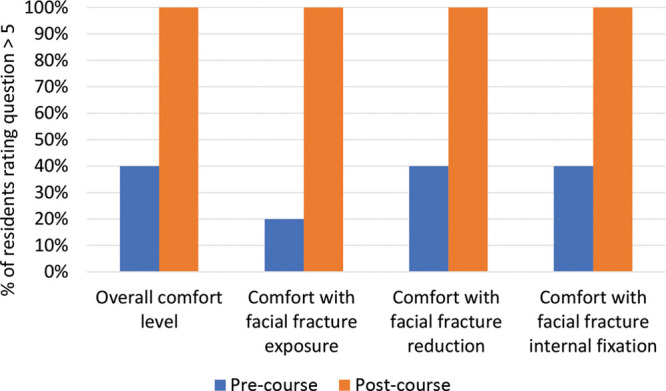
Resident response to questionnaire before and after Facial Fracture Education Course. Scale for each question was 1–10, with 10 being very comfortable with that step in operative facial fracture management.

Pre-course, 40% of participating residents rated their comfort with operative management of facial fractures above 5 (range 3–8), compared with 100% (range 6–9) after. Residents rated their comfort with the principles of operative management: exposure, reduction, and internal fixation. Pre-course, 20% were comfortable with exposure and 40% were comfortable with reduction and internal fixation (range 1–8). After the simulation, 100% of residents reported comfortability with each principle (range 6–9) (Fig. 4). Pre-course, 50% of the residents scored above 50% on the in-service assessment. After the course, 100% of residents scored above 50%, with 90% of participants scoring above 80%.

## DISCUSSION

Surgical simulation courses have become crucial for resident training and board certification.^3^ In total, 79% of trainees report that simulation training would be beneficial in maxillofacial surgery.^4^ This study examined the effectiveness of a mandibular and midface fracture course on knowledge and comfort. Resident confidence and comfort in operative steps and in-service examination scores improved. These outcomes suggest the course would address the paucity of craniomaxillofacial trauma exposure in residency.

Cadaveric models enable residents to operate independently, make surgical decisions, and demonstrate judgment without risk of harming patients.^5^ Three-dimensional skull biomodels, even when overlain with soft tissue materials, do not accurately reflect true anatomy.^6^ Our experience with intentional design of osteotome-created complex facial fractures conferred realistic surgical planning, enhanced by real-time pedagogic attending feedback. Our cadaver-based study did not permit residents to practice physical examination maneuvers, assess for airway status, or manage intraoperative complications. These skills may be acquired through operations more frequently encountered by trainees.

Limitations to cadaveric models include expense and limited availability.^7^ All participants in this funded study incurred no financial burden. This course will continue to be offered at no cost to trainees at our institution. Although this corporate-academic partnership model may not be applicable across residency programs, the cost may be offset by institutional funds diverted from less-effective teaching modalities. Cost-effectiveness of surgical simulation tools must continue to be examined.^8^

Our study did not compare the effect of simulation or didactics alone on outcomes, as the goal of this pilot study was to examine the overall effectiveness of the course in a small cohort. Studies in orthopedics,^9^ gynecology,^10^ and urology^11^ have reported greater technical improvement following surgical simulations, compared with didactic lectures. Because implementation of simulations in resident education is likely multimodal, in conjunction with lectures and textbook learning, our study provides insight into effectiveness of a realistic curricular intervention. Our small sample size of 11 precluded meaningful power analysis, but is comparable in size to similar studies.^2,6,12^ This study did not have an outcome measure of intraoperative performance, due to limited institutional case numbers. However, simulation-based training across specialties has been demonstrated to transfer to the operative setting.^13^

## CONCLUSIONS

Our study demonstrates that a facial fracture course composed of didactics and cadaveric simulation improves resident knowledge of facial fractures and increases confidence in operative management. Osteotome-generated cadaveric facial fractures closely simulates clinical scenarios to train surgical residents to expose, reduce, and fixate. Plastic surgery residents at institutions with the capability to support cadaver-based simulations would benefit from this teaching modality.

## Supplementary Material



## References

[1] Onufer EJ Cullinan DR Wise PE Trauma technical skill and management exposure for junior surgical residents – the “SAVE Lab 1.0.” J Surg Educ. 2019;76:824–831. 30595474 10.1016/j.jsurg.2018.12.003 PMC6615483

[2] Christophel JJ Park SS Nogan SJ A facial trauma simulation course for evaluation and treatment of facial fractures. JAMA Facial Plast Surg. 2017;19:464–467. 28594983 10.1001/jamafacial.2017.0313 PMC5710478

[3] Deutsch ES Wiet GJ Seidman M Simulation activity in otolaryngology residencies. Otolaryngol Head Neck Surg. 2015;153:193–201. 26019133 10.1177/0194599815584598

[4] Ahmed N McVicar IH Mitchell DA Simulation-based training in maxillofacial surgery: are we going to be left behind? Br J Oral Maxillofac Surg. 2019;57:67–71. 30595334 10.1016/j.bjoms.2018.11.009

[5] Tavakol M Mohagheghi MA Dennick R Assessing the skills of surgical residents using simulation. J Surg Educ. 2008;65:77–83. 18439524 10.1016/j.jsurg.2007.11.003

[6] D’Souza N Mainprize J Edwards G Teaching facial fracture repair: a novel method of surgical skills training using three-dimensional biomodels. Plast Surg (Oakv). 2015;23:81–86. 26090347 10.4172/plastic-surgery.1000921 PMC4459413

[7] Palter VN Grantcharov TP Simulation in surgical education. CMAJ. 2010;182:1191–1196. 20351120 10.1503/cmaj.091743 PMC2917931

[8] Henriksen K Rodrick D Grace EN Brady PJ Challenges in health care simulation: are we learning anything new? Acad Med. 2018;93:705–708. 28817431 10.1097/ACM.0000000000001891

[9] Rebolledo BJ Hammann-Scala J Leali A Arthroscopy skills development with a surgical simulator: a comparative study in orthopaedic surgery residents. Am J Sports Med. 2015;43:1526–1529. 25769535 10.1177/0363546515574064

[10] Supramaniam PR Mittal M Davies R Didactic lectures versus simulation training: a randomised pilot evaluation of its impact on surgical skill. Gynecological Surgery. 2018;15:21.

[11] Grober ED Hamstra SJ Wanzel KR The educational impact of bench model fidelity on the acquisition of technical skill: the use of clinically relevant outcome measures. Ann Surg. 2004;240:374–381. 15273564 10.1097/01.sla.0000133346.07434.30 PMC1356416

[12] Froelich JM Milbrandt JC Novicoff WM Surgical simulators and hip fractures: a role in residency training? J Surg Educ. 2011;68:298–302. 21708367 10.1016/j.jsurg.2011.02.011

[13] Dawe SR Pena GN Windsor JA Systematic review of skills transfer after surgical simulation-based training. Br J Surg. 2014;101:1063–1076. 24827930 10.1002/bjs.9482

