# Supplementary material for: Surgical Simulation Course for Facial Fracture Education
Source: Plast Reconstr Surg Glob Open. 2021 Jan 25;9(1):e3353. doi: 10.1097/GOX.0000000000003353 (PMC7859082; doi:10.1097/GOX.0000000000003353)
Supplement: Supplementary file 1 [file gox-9-e3353-s001.pdf]

Fracture Course Posttest:

Name:

(1) How comfortable in general do you feel now with facial fractures? (1-10 with 10 being most)

(2) Do you feel that your residency training would benefit having a required annual course? (1-10)

(3) Rate your comfort level with facial fracture exposure. (1-10)

(4) Rate your comfort level with facial fracture reduction. (1-10)

(5) Rate your comfort level with facial fracture internal fixation. (1-10)

(6) Isolated orbital fractures most commonly occur in which of the following bones?

A) Ethmoid
B) Frontal
C) Lacrimal
D) Maxillary
E) Zygomatic

(7) Which of the following concomitant fractures is most likely to affect the perceived reduction when performing open reduction and internal fixation of a zygomaticomaxillary complex fracture?

A) Anterior maxillary wall comminution with loss of bone
B) Articular tubercle of the zygomatic arch
C) Le Fort I fracture
D) Naso-orbito-ethmoid fracture
E) Orbital floor blowout fracture

(8) Which of the following tooth root apexes is at greatest risk for damage during a Le Fort I osteotomy for maxillary advancement?

A) Bicuspid
B) Canine
C) Central incisor
D) First molar
E) Lateral incisor

(9) A 23-year-old male pitcher is struck in the face with a baseball during practice and sustains an isolated blowout fracture of the medial orbital wall. A transcaruncular approach is chosen for exposure. After incising the medical conjunctiva, dissection is best carried out between which of the following two structures to reach the posterior lacrimal crest?

A) Inferior rectus muscle and capsulopalpebral fascia
B) Lockwood ligament and inferior oblique muscle
C) Medial canthal ligament and lacrimal duct
D) Medial orbital septum and Horner muscle
E) Whitnall ligament and medial rectus muscle

(10) A 25-year-old man comes to the office for treatment of malocclusion 6 days after he was involved in an altercation. Physical examination shows right-sided facial swelling. X-ray study shows an unfavorable fracture through the angle of the right mandible. No other associated injuries are noted. Which of the following is the most appropriate treatment?

A) Closed reduction and maxillomandibular fixation
B) Open reduction and maxillomandibular fixation
C) Open reduction and rigid fixation
D) Open reduction, wire fixation, and maxillomandibular fixation
E) Observation only

(11) Proper reduction of an isolated zygoma fracture requires reduction and realignment of which of the following?

A) Zygomaticofrontal suture, zygomaticomaxillary buttress, and infraorbital rim
B) Zygomaticofrontal suture, zygomaticomaxillary buttress, and orbital floor
C) Zygomaticofrontal suture, zygomaticonasal suture, and infraorbital rim
D) Zygomaticomaxillary buttress, infraorbital rim, and nasomaxillary buttress
E) Zygomaticomaxillary buttress, orbital floor, and alveolus
